# Results of a novel screening tool measuring dietary sodium knowledge in patients with chronic kidney disease

**DOI:** 10.1186/s12882-015-0027-3

**Published:** 2015-03-31

**Authors:** Julie A Wright Nunes, Cheryl A M Anderson, Jane H Greene, Talat Alp Ikizler, Kerri L Cavanaugh

**Affiliations:** Department of Internal Medicine, Division of Nephrology, University of Michigan Health System, Simpson Memorial Building, Room 311 102 Observatory, Ann Arbor, Michigan 48109 USA; San Diego, Division of Preventive Medicine, University of California, San Diego, CA USA; Vanderbilt University Medical Center, Division of Nephrology, Nashville, TN USA; Vanderbilt University Medical Center, Vanderbilt Center for Kidney Disease, Nashville, TN USA

**Keywords:** Patient knowledge, Dietary sodium, Kidney disease, Survey

## Abstract

**Background:**

Reducing dietary sodium has potential to benefit patients with chronic kidney disease (CKD). Little research is available defining dietary sodium knowledge gaps in patients with pre-dialysis CKD. We designed a brief screening tool to rapidly identify patient knowledge gaps related to dietary sodium for patients with CKD not yet on dialysis.

**Methods:**

A Short Sodium Knowledge Survey (SSKS) was developed and administered to patients with pre-dialysis CKD. We also asked patients if they received counseling on dietary sodium reduction and about recommended intake limits. We performed logistic regression to examine the association between sodium knowledge and patient characteristics. Characteristics of patients who answered all SSKS questions correctly were compared to those who did not.

**Results:**

One-hundred fifty-five patients were surveyed. The mean (SD) age was 56.6 (15.1) years, 84 (54%) were men, and 119 (77%) were white. Sixty-seven patients (43.2%) correctly identified their daily intake sodium limit. Fifty-eight (37.4%) were unable to answer all survey questions correctly. In analysis adjusted for age, sex, race, education, health literacy, CKD stage, self-reported hypertension and attendance in a kidney education class, women and patients of non-white race had lower odds of correctly answering survey questions (0.36 [0.16,0.81]; p = 0.01 women versus men and 0.33 [0.14,0.76]; p = 0.01 non-white versus white, respectively).

**Conclusions:**

Our survey provides a mechanism to quickly identify dietary sodium knowledge gaps in patients with CKD. Women and patients of non-white race may have knowledge barriers impeding adherence to sodium reduction advice.

**Electronic supplementary material:**

The online version of this article (doi:10.1186/s12882-015-0027-3) contains supplementary material, which is available to authorized users.

## Background

Research shows that dietary sodium reduction results in significant health benefits in targeted and vulnerable populations. In patients with chronic kidney disease (CKD) reducing dietary sodium prevents cardiovascular events and reduces hypertension and proteinuria associated with kidney failure [1-3]. Prevalence of kidney disease across the world is estimated at 13% or higher [4-7]. Thus, programs that achieve sodium reduction have potential to benefit a significant portion of the population [8,9]. Despite guidelines to limit daily sodium intake [10-13], consumption of dietary sodium in patients with CKD remains high [14].

Patients face numerous challenges to reducing dietary sodium. These include social, economic, health system and individual-patient barriers [15]. Although national campaigns temporally raise consumer awareness about the relationship between high dietary sodium and ill health effects, gains in awareness have not proven to be sustainable over time. Moreover, the percentage of the general population actively trying to limit dietary sodium has never reached over 33% [16]. A recent review suggests that education and provider counseling are critical to help patients with chronic conditions optimize dietary behaviors. However short patient-provider encounters and limited resources hinder this effort [17]. Moreover, patients on dialysis indicate that even after being counseled they do not feel they have the necessary and specific knowledge about how to translate dietary recommendations into day-to-day food selections [18,19].

Little research has been done to identify dietary-specific knowledge barriers in patients with kidney disease who are not yet on dialysis [15]. For example, have patients been encouraged to adhere to dietary sodium limits, and if so, do patients understand their ‘limits’ of intake? Do patients understand that most dietary sodium consumed comes from sources inherent in cooking or using processed foods, and not necessarily applications of seasoning at the table? Is there opportunity to address potential confusion patients face in making diet choices, especially given other comorbid conditions requiring further restrictions related to fat and carbohydrate intake? This information could be of great benefit to the clinician to identify important education opportunities. A survey on such topics could even be used to screen for patient knowledge gaps early in disease and tailor dietary counseling at the point of care or to develop and assess patient-centric programs aimed at improving dietary behaviors in the future.

We designed and implemented a survey tool to quickly identify dietary sodium knowledge gaps in patients with CKD. Prior surveys in kidney disease have found associations between low overall disease knowledge and individual patient characteristics [20,21]; therefore we examined if patient characteristics were associated with lower sodium knowledge scores as measured by our survey. Our survey was designed as a brief screening tool to quickly identify broad knowledge gaps and prompt tailored counseling at the point of care.

## Methods

The 3-item Short Sodium Knowledge Survey (SSKS) (See Additional file 1) was developed after examining published literature reporting on dietary sodium knowledge in patients with kidney disease. The majority of research appeared focused on the dialysis population [22-25]. We obtained content input from experts in nephrology (3), cardiovascular epidemiology (1) and renal nutrition (1). In a cross-sectional study design, we also collected patient demographic information (e.g. sex, age, race) and health literacy using the Rapid Estimate of Adult literacy in Medicine (REALM) [26]. One of the questions asked patients if they knew their daily dietary sodium limit, and we based ‘correct response’ using the National Kidney Foundation Kidney Disease Outcomes Quality Initiative Guidelines on this topic [12]. Lastly, we asked patients about whether they received counseling specific to limiting dietary sodium (yes/no), whether they took action if they had received counseling, and whether they could identify their recommended dietary sodium intake limit.

The survey was performed as part of a larger study assessing patient knowledge about many aspects of kidney disease, communication, and kidney care [27]. Initial participants (approximately the first 10–20) were asked to comment on questions to ensure interpretability. The survey was designed at a 6th grade reading level (using Flesch-Kincade reading grade level). The setting included nine general nephrology clinics within one academic center. All adult patients with established chronic kidney disease [28] seen within these clinics who were not new patients were invited to participate using convenience sampling. Specifically, patients with either “kidney damage OR an estimated Glomerular Filtration Rate (eGFR) < 60 ml/min/1.73 m2 for ≥ 3 months” [28] were included. Patients who were receiving dialysis or who had a kidney transplant or those who could not speak or understand English were excluded. If lower literacy was identified during the REALM assessment the survey was administered as an interview to the patient in a private room by trained research staff. The Vanderbilt Institutional Review Board approved the study and informed consent was obtained from all participants. Participants were offered a small monetary compensation.

Descriptive statistics are reported as mean (standard deviation, SD) for continuous variables, or frequency (%) for categorical variables. Severity of CKD stage was determined using laboratory serum creatinine and urinary protein measurements abstracted from the medical record [28]. The 4-variable Modification of Diet in Renal Disease equation was applied to calculate estimated glomerular filtration rate (eGFR) [29]. We also asked participants if they had a diagnosis of hypertension and if they had attended a kidney disease education class. We used self-reported hypertension in analysis as prior research has shown good accuracy in patient self-report of this diagnosis [30]. The education class is provided on site by a renal dietician educator covering diverse kidney disease topics, including medical nutritional counseling. We report participant responses to each of the sodium knowledge questions as frequency (%). We also report the frequency of patients who responded correctly to the all three of the questions.

We performed logistic regression to examine the association between sodium knowledge and patient characteristics. Economic status may influence behavior related to purchasing certain foods, although direct impact on objective dietary sodium knowledge is less clear. If there is an impact it may be mediated through lower health literacy and less education; therefore, we included education status and health literacy in our analyses. Characteristics of patients who answered all three SSKS questions correctly were compared to those who did not. Each question addressed a unique but important area of sodium knowledge, providing the rationale to examine the three questions as a summary score. Logistic regression was adjusted for age, sex, race, education, health literacy, stage of CKD, self-reported hypertension and attendance in a kidney education class and associations with a p value of ≤ 0.05 were deemed statistically significant. STATA version 10.0 (College Station, Texas) was used for all statistical analyses.

## Results

Baseline characteristics for the 155 participants are presented in Table 1. 314 patients were approached to take our survey. 148 declined and 11 withdrew after consent due to time constraints, feeling ill or other reasons. In comparing age and sex between initial survey responders and non-responders we found no differences in sex. Non-responders were slightly older compared to responders (mean (SD) age 61.5 (15.5) years non-responders versus 56.6 years (15.3) in responders; p = 0.004). The mean (SD) age of patients participating was 56.6 (15.1) years and 80% had moderate to severe CKD with an eGFR of ≤ 60 ml/min/1.73 m^2^. Eighty-four (54.2%) were men, 119 (76.8%) were white and 142 (92.8%) had a high school graduate or higher level education. Thirty-six patients (23.2%) were categorized as non-white for analysis and this comprised 30 patients reporting they were African American, 2 Asian and 4 reporting as ‘other’. Over 20% had limited health literacy assessed as ≤ 9th grade reading level and the majority self-reported a diagnosis of hypertension. Twenty-four patients (15.7%) reported attending the kidney disease education class.

**Table 1:** **Patient baseline characteristics, N = 155**

**Characteristics**	**Mean (S.D.) or (%) – all participants**	**Mean (S.D.) or (%) – participants answering all survey questions correct**
Age, years	56.6 (15.1)	56.1 (14.5)
Sex, male	54.2%	58.8%
Race, white	76.8%	84.5%
H.S. Graduate or more years of education	92.8%	93.8%
Health literacy ≤ 9th grade	22%	17.5%
Self-reported hypertension	88.4%	90.7%
CKD stage*		
1-2	20.7%	21.7%
3	47.7%	46.4%
4-5	31.6%	32.0%
Attended kidney disease education class with renal dietitian	15.7%	17.5%

*Defined using National Kidney Foundation KDOQI guidelines [26].

One-hundred thirty-three of the 155 respondents (86.4%) reported receiving nutritional counseling from their providers to reduce dietary sodium intake; the majority of these patients said they followed this advice (97%). Forty-three percent of patients (43.2%) knew their sodium intake should be less than 2400 mg per day. The majority of patients correctly responded to 3-item SSKS when examined individually. However, only 97 patients (62.6%) correctly answered all SSKS questions as an overall summary of sodium knowledge (Table 2). For comparison we also report overall characteristics of patients correctly responding to all of the 3-item SSKS, and this is shown in the third column of Table 1.

**Table 2:** **Response results for survey, N = 155**

**Question/topic**	**Response n (%)**
Reported being told by provider to restrict sodium intake	133 (86.4)
Of those told to restrict sodium intake, number reporting they followed this nutritional counseling	129 (97.0)
Knowledge of daily sodium intake limit	67 (43.2)
*Correctly identified higher sodium containing food	130 (83.9)
*Correctly identified a way to decrease sodium intake	128 (82.6)
*Correctly recognized most sodium intake comes from processed/packaged foods	116 (74.8)
Correctly answered all 3 items of SSKS survey questions	97 (62.6)

*Designates 3 items of Short Sodium Knowledge Survey, SSKS.

Unadjusted analysis examining the relationship between the SSKS and individual patient characteristics revealed that non-white patients had 0.32 odds ratio ([95% C.I. 0.15,0.69]; p < 0.01) of answering all survey questions correctly compared to patients who were white. In an adjusted analysis including age, sex, race, education, health literacy, self-reported hypertension, CKD stage, and kidney education class, women and patients of non-white race had lower odds ratios of correctly answering all SSKS questions (0.36 [0.16,0.81]; p = 0.01 women versus men and 0.33 [0.14,0.76]; p = 0.01 non-white versus white, respectively). In addition, we noted a trend in that patients who reported a diagnosis of hypertension had three times higher odds ratio (3.16 [0.94, 10.59], p = 0.06) of answering all questions correctly compared to patients who did not report hypertension, although this was not statistically significant (Table 3).

**Table 3:** **Unadjusted and adjusted odds ratios (OR) from logistic regression analysis of all correct sodium knowledge questions**

**Characteristic N = 155**	**Unadjusted OR [C.I.]; p value**	**Adjusted OR [C.I.]; p value**
Age, years	0.99 [0.97,1.02]; p 0.55	0.99 [0.96,1.01]; p 0.35
Sex		
Male	Reference	Reference
Female	0.61 [0.32,1.18]; p 0.14	0.36 [0.16,0.81]; p 0.01
Race		
White	Reference	Reference
Non-white	0.32 [0.15,0.69]; p <0.01	0.33 [0.14,0.76]; p 0.01
Formal educational attainment		
< H.S. Graduate	Reference	Reference
≥ H.S. Graduate	1.44 [0.42, 4.95]; p 0.56	0.67 [0.15,2.98]; p 0.59
Health literacy		
<9th grade	Reference	Reference
≥9th grade	2.00 [0.92, 4.33]; p 0.08	2.27 [0.86,5.98]; p 0.10
Self-reported hypertension		
No hypertension	Reference	Reference
Hypertension	1.79 [0.67,4.82]; p 0.25	3.16 [0.94,10.59]; p 0.06
CKD stage		
Stages 1-2	Reference	Reference
Stage 3	0.81 [0.34, 1.93]; p 0.64	0.95 [0.32.2.86]; p 0.93
Stages 4-5	0.90 [0.36, 2.29]; p 0.83	1.05 [0.33,3.37]; p 0.93
Kidney disease education class		
Did not attend	Reference	Reference
Did attend	1.49 [0.58,3.84]; p 0.41	1.67 [0.57,4.92]; p 0.35

As an additional exploratory analysis, we also examined if patient self-report of diabetes mellitus was associated with correctly answering all SSKS questions. We found no significant association. Including this in the fully adjusted model did not change which characteristics were statistically significant nor the odds ratios significantly. We also explored whether a one-time check-in systolic blood pressure was associated with correctly answering SSKS questions and found no significant association.

## Discussion

Time and resource constraints during busy patient-provider encounters demand both efficient and effective means to identify and address the medical needs of our patients with chronic conditions. Our survey provides an efficient way to screen for dietary sodium knowledge gaps in patients and identify those in need of supplementary educational support at the point of care. Our survey also identifies gaps in patient knowledge about basic nutritional concepts important to dietary adherence. More than half of participants were unable to identify a recommend upper limit of intake. Furthermore, in this group of patients who might be considered the most well-primed to have good overall sodium knowledge (i.e. all were seeing a kidney specialist, had an established diagnosis of CKD, and most diagnosed with hypertension), nearly 40% failed to correctly answer all questions in our brief survey. We also identified patient characteristics associated with low sodium knowledge, suggesting disparities in dietary sodium knowledge by gender and race.

Research shows that dietary interventions focused on sodium reduction lower blood pressure, decrease extracellular fluid volume and reduce proteinuria in patients with kidney disease [31]. Not all elements implemented within these interventions can be easily translated into services supported by our current health care system. Specifically, current routine practice for patients in clinical settings cannot directly supply patients with low sodium foods nor do they routinely have the resources to support frequent and close monitoring and dietary counseling day-to-day. Identifying areas of low sodium knowledge efficiently could assist in focusing patient-provider discussion in problem areas for patients. It could help facilitate discussion about ‘real-world’ food selections and healthy dietary habits in a way that is feasible for busy clinical settings.

Interestingly, in our study, 97% of patients who received advice to cut back on sodium intake reported doing so. A comprehensive review spanning three decades of research on patient adherence cites difficulty trying to generalize patient adherence but generally it “is to be expected in 30-50% of all patients, irrespective of disease, prognosis, or setting” [32]. In comparison our findings seem strikingly high. Perhaps patients in our study were more apt to report following advice in the setting of chronic kidney disease and often co-existing hypertension. A recent meta-analysis examining the impact of dietary counseling in cardiovascular disease showed provider-led counseling improves patient behaviors and reduces cardiovascular risk compared to usual care [33]. This supports the importance of provider-messaging. However, it is concerning that provider advice to “cut back on sodium” did not appear to translate into higher patient knowledge as measured by the overall sodium knowledge in the SSKS.

In addition, only 43% of patients correctly identified their recommended daily sodium limit. It is unclear if this is because providers did not discuss a sodium limit in terms of a specific number or whether the information that was provided was not clear. Studies show knowledge of specific health-related goals is associated with improved clinical outcomes; patients who are aware of their blood pressure goal are more likely to achieve good blood pressure control versus those not aware [34]. This may be mediated by more education for these patients in self-care areas like dietary sodium restriction and perhaps offers one reason for the trend we observed of higher knowledge in patients reporting a diagnosis of hypertension. Thus, there may be an important opportunity to help some patients further optimize their sodium intake by providing them with concrete information about recommended daily limits, i.e. in terms of a specific number.

We also found that there is still opportunity to help patients identify which foods contain higher content of sodium relative to one another. This is important in day-to-day food selections. Twenty-five percent of patients did not know that processed/packaged foods provide the highest source of sodium intake compared to other foods. Building interventions that can help patients identify higher sodium foods and possibly select healthier alternatives is not easy [2]. Pilot studies provide encouragement. Personal data assistant (PDA) technology helps patients track sodium intake and reduces inter-dialytic fluid consumption and weight gain in dialysis [35]. Similar tracking and support mechanisms could be used in patients not yet on dialysis serving as an adjunct to initial dietary counseling from providers.

Two patient characteristics were associated with lower SSKS scores, namely non-white race and female gender. Findings in other kidney disease knowledge surveys found similar results [20,21,36]. Some attribute lower scores on knowledge surveys to education or health literacy levels. However, our analysis included adjustment for both of these factors as well as adjustment for a local specific kidney education class. Because of limited sample size we were not able to stratify by race further, but the majority of the patients reporting as non-white race in our study did report they were African American (83%). There is an association between African American race and increased salt-sensitivity [37] making our findings even more concerning. In addition, a study examining sodium intake in patients on maintenance dialysis found that women were more likely to report problems managing diet and had difficulty adhering to their hemodialysis regimen overall [19]. Research in diabetes shows that women are significantly more likely to perform grocery shopping and prepare meals in households [38]—which has further potential to impact dietary behaviors in all who live within. It is not clear if our observations were due to differences in counseling patients received or other characteristics we did not measure. Our research suggests a need for future studies to explore reasons behind the differences and to develop gender and culturally sensitive programs to address these disparities.

We also identified a statistical trend between higher health literacy and higher odds of scoring all SSKS items correctly. Prior work reveals that knowledge is associated with health literacy status in patients who have kidney disease [39]. Moreover, patients with limited health literacy are at higher risk for poor health outcomes [40] and have less access to optimal treatments for kidney failure [41]. The trend we observed seems to reflect the critical importance of ensuring patient-provider dialog and education are tailored to meet the individual communication needs of our patients.

## Conclusions

Although we identified important and modifiable knowledge gaps through the use of our survey, this study has limitations. It is a cross-sectional qualitative study. We cannot imply causation. Our findings still are important in that we identified patient knowledge gaps that can be easily integrated into individualized counseling sessions for patients. Another limitation is that the guidelines for sodium intake may differ depending on which guidelines are applied [12]. This could have impacted patient response to our question asking them to identify their daily sodium limit--highlighting a need for a consistent message when conveying sodium intake goals from all providers. Third, our participating patients were recruited by convenience sampling within clinics at one academic institution. Our population may not represent the population of patients with CKD across the entire U.S. But comparing patient characteristics of our sample to the United States Renal Data System, we found demographic characteristics are strikingly similar [42]. Convenience sampling could also introduce bias. Perhaps only patients who felt they knew more about dietary sodium actually chose to take the survey. If this were the case we would expect bias towards the null. Yet we still observed significant associations highlighting important areas for future research and intervention development. Finally, our survey was not designed as a uni-dimensional comprehensive assessment of dietary sodium knowledge in patients: based on our prior review of the literature [15], this remains an important opportunity for future research in patients with CKD, not yet on dialysis.

Despite limitations there are practical strengths of our study. Our sodium survey provides a way to quickly assess knowledge gaps about dietary sodium in patients with pre-dialysis CKD. It could be used to identify individual patient education needs in a clinical encounter or to monitor progress after nutritional interventions have been provided. We also found that patients need more specific information about how to translate general advice to reduce dietary sodium into concrete day-to-day eating behaviors. Creating a unified message about sodium reduction and intake goals across provider encounters may offer benefit in this arena.

In conclusion, we identified important and modifiable knowledge gaps through the use of a survey that briefly assesses patient knowledge about dietary sodium. Our findings suggest that women and patients of non-white race may have knowledge barriers that impede adherence to sodium reduction advice. Interventions need to be developed to address these barriers and support the educational needs for all patients with chronic kidney disease.

## Additional file

Additional file 1:
**Supplement-Patient survey questions.** Asterisks and bold lettering denote our 3-item Short Sodium Knowledge Survey (SSKS).
