# Supplementary material for: Results of a novel screening tool measuring dietary sodium knowledge in patients with chronic kidney disease
Source: BMC Nephrol. 2015 Mar 31;16:42. doi: 10.1186/s12882-015-0027-3 (PMC4387682; doi:10.1186/s12882-015-0027-3)
Supplement: Additional file 1: — Supplement-Patient survey questions. Asterisks and bold lettering denote our 3-item Short Sodium Knowledge Survey (SSKS). [file 12882_2015_27_MOESM1_ESM.doc]

Supplement-Patient survey questions. Asterisks and bold lettering denote our 3-item Short Sodium Knowledge Survey (SSKS).

***1. Which food item has the most sodium?**

**□ 1 medium sized apple**

**□ 2 ounces of lunchmeat (for example, turkey breast) (SCORED AS CORRECT)**

**□ 1 banana**

**□ 1 plain baked potato**

***2. What is the best way to cut back on sodium you eat?**

**□ Trim visible fat from meats**

**□ Eat more fresh vegetables instead of canned vegetables (SCORED AS CORRECT)**

**□ Substitute skim milk for whole milk**

**□ Drink more fresh squeezed orange juice instead of canned juice from concentrate**

***3. Where does most of the sodium that people eat come from?**

**□ salt added at the table when eating**

**□ processed / packaged foods (SCORED AS CORRECT)**

**□ soda pop**

**□ plain frozen vegetables**

4. Has a health care provider ever told you to cut down on the amount of sodium (or salt) you eat?

□ Yes □ No

4a. If yes, did you make changes to your diet to cut down on sodium / salt?

□ Yes □ No

5. The amount of sodium that you eat every day should be less than what number?

□ I do not have a number for limiting sodium that I eat

□ 2400 milligrams/day [12]

□ 3400 milligrams/day

□ 5000 milligrams/day
